# Are Human Learners Capable of Learning Arbitrary Language Structures

**DOI:** 10.3390/brainsci13020181

**Published:** 2023-01-21

**Authors:** Yu-Leng Lin

**Affiliations:** Department of Foreign Languages and Literature, Feng Chia University, Taichung 407102, Taiwan; yuleng.lin@mail.fcu.edu.tw

**Keywords:** artificial grammar learning, harmony, phonetic naturalness, phonology

## Abstract

The artificial grammar learning paradigm is a classic method of investigating the influence of universal constraints on shaping learning biases on language acquisition. While this method has been used extensively by linguists to test theoretical claims in generative grammar, one of the most prevalent frameworks in language acquisition, several studies have questioned whether artificial grammar learning reflects language acquisition enough to allow us to use it to draw inferences about the validity of universal constraints, particularly those arising from phonetic naturalness. The current study tests whether artificial grammar learning shows the effect of one robust phonetic naturalness constraint: the restriction on nasal harmony patterns arising from the sonority hierarchy. Nasal harmony is of particular interest because it is one of the few types of harmony that occurs between consonants and vowels, which is an under-researched topic. The results, contrary to the skeptical concerns, showed that participants (*n* = 120) could learn an artificial grammar involving a natural pattern, but could not learn one corresponding to an arbitrary/phonetically unmotivated pattern in the same way or to the same degree. This study contributes epistemic support to the large body of work using artificial grammar experiments to test phonological operations.

## 1. Introduction

Phonological research seeks to distinguish different causes of the patterning of sounds: Which arise from constraints on learnability provided by human biology? Which represent epiphenomena arising from articulatory or acoustic factors? Which are simply the residue of historical sound change? These questions have been examined using typological surveys, formal theoretical investigations, computational modeling and experimental methods [1]. One paradigm which has been particularly fruitful in distinguishing these types of patterns, according to its proponents, is artificial grammar learning. The virtue of the artificial grammar learning paradigm is that it allows us to test whether learning of putatively “impossible” patterns is indeed difficult or impossible. If a pattern predicted to be excluded by biological constraints is found learnable in an artificial grammar experiment, this raises the question whether the theory of biological constraints needs to be revised to account for the possibility of acquiring patterns predicted to be impossible, or indeed whether learning biases discovered in laboratory settings should be modeled within the theory at all.

One phonological pattern that presents a test case for a theory of learnability is nasal harmony. Nasal harmony refers to a phenomenon in which a segment bearing nasality (called the trigger) spreads its nasality to other segments (called targets). Some segments (called blockers or opaque segments) do not undergo nasalization, and furthermore, stop its spread. Other segments (called transparent) do not undergo nasalization but do not stop its spread (See [2] for more discussion, albeit within the context of vowel harmony). Like all harmony phenomena, learning nasal harmony requires learners to be able to identify the harmonizing segments, either consciously or unconsciously. The kind of harmony at issue in this paper involves the spreading of nasalization from a [nasal] feature to target segments, which may be either consonants or vowels. For example, in Johore Malay, a nasal stop, the trigger, spreads its nasality to targets rightward, namely vowels, laryngeals and glides (e.g., /mãʔ̃ãp/ ‘pardon’) until encountering a blocker, namely a liquid or obstruent (e.g., /pəŋãw̃ãsan/ ‘supervision’) [3]. In the case of /pəŋãw̃ãsan/, nasalization originating in the trigger /ŋ/ spreads rightward to all targets but does not spread to, or beyond, the blocker /s/. This indiscriminate choice of targets is unusual among harmony phenomena, which typically constrain themselves to affecting either consonants or vowels (see [4] for more discussion of vowel–consonant harmony, vowel harmony and consonant harmony). (In addition to the type of nasal harmony examined in this paper, there is another variety of nasal harmony, which is non-local and affects only consonants. Specifically, nasal stops act as a trigger for the change of voiced stops and oral sonorant consonants into nasal stops [5]).

The aspect of nasal harmony that is of interest in this paper is the identity of the segments which function as targets and blockers. In general, whether a segment functions as a target or a blocker appears to be a function of its sonority. One theoretical construct that attempts to capture this generalization is the nasalized segment hierarchy. The nasalized segment hierarchy is used to account for the fact that, cross-linguistically, (i) a segment’s likelihood of undergoing nasalization in nasal harmony corresponds to its position in the sonority hierarchy [5] and (ii) a segment’s likelihood of blocking nasalization in nasal harmony is inversely related to its position in the sonority hierarchy [5]. It is debated whether the third type of relationship a segment can have with nasal harmony, namely transparency, should be explained by the nasalized segment hierarchy, given that the class of transparent segments varies from language to language, sometimes including obstruents and other times including sonorants. It is also debated whether transparent segments are truly transparent phonologically or whether their lack of nasalization in a nasal harmony environment is a phonetic implementation detail (see further discussion in [5,6,7,8,9,10,11,12,13,14]). As a result of these open questions, only targets and blockers will be included for the remainder of this paper. In particular, we will be concerned exclusively with one side of the nasalized segment hierarchy, i.e., the relationship of blocking to sonority. To state this relationship in terms of an implicational universal, we might say: higher sonority segments acting as blockers for nasalization in nasal harmony implies that lower sonority segments act as blockers as well. 

One question then arises: Is the sonority hierarchy violable or reversible? In other words, is it possible for humans to learn a pattern of nasal harmony that violates the sonority hierarchy? If so, this finding would cast doubt on the validity of the sonority hierarchy as a phonological universal, assuming that restrictions on learnability are the source of linguistic universals. Along the same lines, some scholars have stated that the findings of artificial grammar learning experiments can teach us about universal grammar (UG [15]) because these experiments are designed to mimic features of child language acquisition, where participants are exposed only to limited data without correction. Many artificial grammar studies [2,16,17,18,19,20,21,22,23,24,25,26] follow this working assumption.

Another working assumption of the artificial grammar learning paradigm is that participants should be able to be trained to acquire a variety of artificial grammars in an experimental setting regardless of their native language(s). There remains, however, the question of how varied the class of learnable artificial grammars is. In other words, can participants learn grammars which are very much unlike those of natural languages? In phonology, for example, we ask whether participants can learn ‘unnatural’ sound patterns, i.e., patterns which are typologically unattested and phonetically unmotivated. If participants are able to learn *any* given pattern, either (a) the assumption that a theory of grammar should exclude these ‘unnatural’ patterns must be dropped, or (b) the mechanisms involved in artificial grammar learning are in some way different from those involved in natural language acquisition, which would render the paradigm of little utility in examining the nature of language.

One way to see whether we must confront this dilemma is to design an artificial grammar which exhibits a flagrantly *unnatural* process: nasal harmony governed by a reversed sonority hierarchy. If participants could not learn this grammar’s pattern, or if it were more difficult for them to learn the pattern, this could be used as evidence to support the claim that only phonetically natural patterns (i.e., the patterns obeying the sonority hierarchy) are learnable. However, if participants could learn this reversed pattern in some way, it could be used to argue that phonological theory should be modified to accommodate the learnability of anti-sonority hierarchy patterns. We found, however, that the unnatural pattern was not learnable in a laboratory setting, at least not in the same way or to the same degree as the natural pattern, lending evidence to the claim that such unnatural patterns are unlearnable or disfavored given the human cognitive and/or physiological endowment.

## 2. Methods

### 2.1. Language Populations: Taiwan Southern Min

The language population chosen for this study consists of speakers of Taiwan Southern Min (S. Min). The rationale for choosing speakers of S. Min arises from one of the assumptions made by experiments testing the bias towards UG structures in phonological artificial grammar learning. The assumption is that the target language should show a contrast in the feature of interest (in this case, nasality), but that it should not have a productive phonological process related to the process of interest (here, nasal harmony across word boundaries). The reason for this restriction is that, if the target phonological process were already productive in the target language, then any learning biases found could be ascribed to language-specific influence rather than to the influence of language universals. S. Min meets both of these criteria: it has nasalized vowels and nasal consonants but it does not exhibit productive nasal harmony [27]. (Nasal consonants are in complementary distribution with voiced oral stops, with oral stops appearing before oral vowels and nasals appearing before nasal vowels. Stimuli were designed to avoid confounding factors arising from this allophonic variation. Some phenomena in S. Min could be analyzed as nasal harmony (e.g., /bin^33^ + a^53^/ /tsaj^31^/ → [mj̃ã^33^ tsaj^31^] ‘tomorrow’), but all such examples are lexicalized [27,28]). That is, if S. Min speakers can learn nasal harmony patterns, this likely reflects the influence of the human language faculty, given that nasal harmony across word boundaries is absent from S. Min phonology. Consider the phonemic inventory of S. Min, which is presented in Table 1 and Table 2. 

There are 20 consonants in total in S. Min: 10 stops, 3 nasals, 3 fricatives, 2 affricates, and 2 approximants. S. Min has a three-way laryngeal contrast: voiced, voiceless unaspirated, and voiceless aspirated. There are six oral vowels and four nasal vowels. The syllable structure of S. Min is (C)(G)V(C), although only nasals /m, n, ŋ/, voiceless stops / p, t, k, ʔ/ and approximants /j, w/ can fill the coda position. S. Min has seven contrastive tonal categories, namely 55 (HH), 24 (LM), 51 (LH), 21 (ML), 33 (MM), 21 (mid checked) (A checked tone refers to a syllable with a coda stop [p, t, k, ʔ] and partial tonal neutralization.) and 53 (high checked). The tonal values for the monosyllables concatenated to form the tri-syllabic non-words used in the current study were all high-level tones 55 (HH). Because the tonal values of all stimuli were 55, no tonal diacritics are marked for ease of reading and to avoid distracting from the nasalization diacritics that mark the phenomenon of interest in the current study.

### 2.2. Design

Participants were divided into two trained groups and two control groups. One trained group and one control group were presented with a grammar conforming to natural patterns. The other two groups, one trained and one control, were presented with a grammar conforming to unnatural patterns. The two trained groups were presented with an exposure phase in which they were exposed to nasal harmony phenomena, while the two control groups were presented with an exposure phase in which no nasal harmony phenomena were present. Specifically, the trained groups were presented in the exposure phase with tri-syllabic items where the first syllable always contained a trigger or non-trigger. These items were organized in pairs, where one item contained no nasalization and the other contained nasalization. In the items which contained nasalization, nasalization spread according to the rules given by one of two nasal harmony grammars, which will be explained below. In the control groups, however, the exposure phase involved items with a di-syllabic word structure. These di-syllabic items were created by removing the first syllable of the tri-syllabic items used for the trained group, which contained the trigger or non-trigger, following the design of [23]. Because the first syllable was removed, no rightward harmony at all occurred in the exposure phase stimuli for the control groups. As a result, items were not presented in pairs to the control groups, because the paired items would have been indistinguishable without the harmony-triggering initial syllable. Otherwise, the design of the test items was identical for trained and control groups. The purpose of including control groups in addition to the trained groups was to ascertain whether any learning effects found were indeed the result of exposure to the harmony phenomenon in the exposure phase or, alternatively, if they could instead be attributed to pre-existing language-specific bias.

All groups received the same test items. All groups were given a post-test in which participants were presented with a nasalized monosyllable and its oral counterpart and were asked to determine which one was nasalized, with the purpose of verifying that participants were able to distinguish nasalized from non-nasalized syllables. The monosyllables chosen were those which were concatenated to create the tri-syllabic stimuli for the exposure and test phases. 

The predictions regarding the generalizability of the natural and unnatural patterns for the trained groups are conceptualized as shown in Table 3.

In Table 3**.**, each row represents one artificial grammar language, one natural and one unnatural. The natural and unnatural grammars examine the implicational universals of the targeting and blocking effects of the sonority hierarchy, namely: (1) blocking by the more sonorous phonological class (a less prototypical blocker, e.g., [w]) implies blocking by the less sonorous phonological class (a more prototypical blocker, e.g., [t, k]), but not vice versa; (2) targeting by the less sonorous phonological class (a less prototypical target, e.g., [t, k]) implies targeting by the more sonorous phonological class (a more prototypical target, e.g., [w]), but not vice versa. These universals predict, therefore, that if participants are exposed to a more prototypical segment (either blocker or target) and then tested with a less prototypical segment, they will be able to generalize from the more prototypical segment to the less prototypical segment, but not vice versa. (This prototypicality can be conceived of either as markedness or as an analogous theoretical construct.) The segments [t] and [w] were always present in both exposure and test phases (see Table 3). The only difference is that the natural group was exposed to [w] as a target and [t] as a blocker (see the ‘exposure’ column in Table 3), and then tested with [k] (see the ‘test’ column in Table 3). On the other hand, the unnatural group was exposed to [t] as a target and [w] as a blocker, and then tested with [k]. If the implicational universal holds, the natural group is expected to be able to generalize from a blocker [t] to a new segment [k], treating [k] as a blocker as well (see the ‘prediction’ column in Table 3), since both [t] and [k] are stops. That is, both [t] and [k] are equally prototypical blockers. However, for the unnatural group, we expect a different result. This group is expected not to be able to generalize from the target [t] to a new segment [k], since targeting by less sonorous segments [t, k] implies targeting by the more sonorous segment [w]; [w], however, was treated as a blocker in the unnatural group. 

### 2.3. Participants

In total, 128 participants were recruited. All were recruited from Feng Chia University in Taiwan. Eight participants in total were excluded from the analysis. Three were excluded because their background questionnaire revealed that they were not S. Min speakers. Five participants failed to distinguish between the pair /ã/ and /a/ in a post-test. The remaining 120 participants were included in the analysis, and were evenly divided into four groups, according to the type of pattern they were exposed to and whether they received training on that pattern, following a 2 × 2 design: naturalness of pattern (natural vs. unnatural) × training effect (trained vs. control groups). The trained group exposed to the natural pattern contained 31 participants, and the trained group exposed to the unnatural pattern contained 29 participants. The other two groups contained 30 participants each. 

All participants had studied a foreign language (English: 120, Japanese: 54, Spanish: 18, French: 18, German: 10, Korean: 10, Russian: 1, Italian: 1). Three of them had been exposed to Hakka. None of these languages has a productive nasal harmony process. The average age of the participants was 20.5. Of the participants, 37 were male and 83 were female. All reported normal hearing, but, as mentioned above, five failed to distinguished nasalized monosyllables from their oral counterparts. The remaining 120 participants received a rate of 99% in the post-test (see Section 2.5), and all remaining participants reported early childhood S. Min exposure. Each participant received compensation based on the time spent participating in the experiment at a rate of 160 NTD/hour. For participants in the control groups, the experiment lasted approximately 40 min on average, and for participants in the trained groups, the experiment lasted approximately 50 min on average. The time taken to complete the experiment differed between control and trained groups because the stimuli for the control groups were di-syllabic items without triggers—that is, no pairs of singular and plural forms were given to the control groups. Since the stimuli used in the control groups did not contain triggers, no training items would show nasal harmony. For this reason, the control groups received no clue about the rule governing the singular-plural alternation. This meant that, in the test phase, they simply had to guess; that is, unless any pre-existing language-specific bias affected the results. Since they did not need to actively search for a rule governing pluralization, they spent less time completing the experiments than the trained groups did. Anomalies did occur for two participants, who always chose to give a single answer (either 1 or 2) throughout the experiment. However, those participants also failed to pass the post-test for distinguishing between the pair /ã/ and /a/. The reason for this is probably that these participants did not concentrate on learning: they simply chose to give the same response no matter what stimuli they listened to.

### 2.4. Stimuli

All stimuli were produced by a male native speaker of S. Min. The experiment design follows a paradigm used extensively in phonological artificial grammar learning [29,30,31]. As mentioned in Section 2.3, a 2 (natural vs. unnatural) × 2 (trained vs. control) design was adopted, where the only difference between trained and control groups lay in the items that they received in the exposure phase.

#### 2.4.1. Control Groups

For the control groups, since no harmony phenomenon was presented in the exposure phase, the exposure phase stimuli consisted of aural di-syllabic forms, as shown in Table 2. No images were necessary to establish a morphological relationship between alternants, as there was no alternation presented. 

The di-syllabic stimuli shown in the training phase to the two control groups tested with the natural and unnatural patterns were identical, as shown in Table 4. The reason that the training items were the same is that, without a trigger, no nasal harmony occurs and therefore there is no alternation to learn. The only difference between the two control groups with respect to the training items is to be found in the number of repetitions of each item: stimuli where the first syllable contained a target were shown twice. If a group would be tested on a grammar where [w] was a target, that group would be shown [wata] twice, but [tawa] once; similarly, the group to be tested on a grammar where [t] was a target would be shown [wata] once, but [tawa] twice. Since the segment serving as a blocker/target was the opposite in both groups (natural: [w] as a target and [t] as a blocker; unnatural: [t] as a target and [w] as a blocker), each of two control groups saw any given stimulus a different number of times in the training phase, but the total number of exposures was the same for both groups.

The test items for the control groups were the same as the ones used for the trained groups. The stimulus design for the trained groups will be detailed in Section 2.4.2.

#### 2.4.2. Trained Groups

For the trained groups, the test phase and the exposure phase used similar stimuli. In both natural and unnatural conditions, participants were exposed to pairs of morphologically related artificial words, each with a singular and plural form. The singular forms were composed entirely of oral segments and the plural forms were marked by nasalization of the initial vowel. This nasalization was also realized on subsequent segments, although the rightward spreading of nasalization was blocked by certain intervening consonants. The classes of consonants which prevented spreading of nasalization (i.e., blockers) were what differed between the groups: in the natural pattern group, e.g., (1), stops were blockers (segments that halt spreading of nasalization) and glides were targets (segments that undergo nasalization); in the unnatural pattern group, e.g., (2), glides were blockers and stops were targets.

(1) Example stimulus for the natural pattern group
  - atawa (singular)
  - ãtawa (plural)

(2) Example stimulus for the unnatural pattern group
  - atawa (singular)
  - ãtãwa (plural)

More specifically, the stimuli were constructed as follows: in both exposure and test phases, the stimuli were of the shape V_1_C_1_V_2_C_2_V_3_. The reasons for selecting stimuli of the specific phonological shape (V_1_C_1_V_2_C_2_V_3_) are twofold. First, a V-initial order was chosen because, if stimuli of a CV-initial shape were used, S. Min allophonic rules would force the C and V to match in specification for [nasal]: i.e., [ta] or [nã] are valid syllables in S. Min, but *[tã] and *[na] are not. To avoid this confounding factor, the first syllable had to be V-initial. Second, tri-syllabic stimuli were chosen because this was the shortest syllable length which would allow us to see the difference in nasal spreading between the natural and unnatural groups. Technically, longer stimuli could have been used, but given that the length of the experiment for the trained groups was already around 50 min, tri-syllabic stimuli were used in order not to exhaust participants.

All syllables carried a high-level tone (55) and conformed to the phonotactics of S. Min. The vowel V_1_ was drawn from one list of segments, the consonants C_1_ and C_2_ from another, and the vowels V_2_ and V_3_ from a third. The initial vowel V_1_ was always [a] in singular forms and [ã] in plural forms, in both exposure and test phases.

The consonants C_1_ and C_2_ were drawn from different sets in the exposure and test phases. In the exposure phase, these consonants were drawn from the set [t, w]. In the test phase, however, these consonants were drawn from the set [k, t, w]. This difference between the set of consonants used in the exposure and test phase stimuli is because we wanted to test whether participants could generalize from rules involving one member of the natural class of stops (i.e., [t]) to another member (i.e., [k]).

The vowels V_2_ and V_3_ were also drawn from different sets in the exposure and test phases. In the exposure phase, these vowels were drawn from the set [a, i]. In the test phase, however, these vowels were drawn from the set [a, e] (or their nasalized counterparts, which we will discuss shortly). The difference between the set of vowels used in the exposure and test phase stimuli is because we wanted to test whether participants could generalize from rules involving members of the natural class of vowels (i.e., [a, i]) to a novel member (i.e., [e]).

The vowels V_2_ and V_3_ appeared in their nasalized forms in both exposure and test phases under certain circumstances. These circumstances were as follows: (i) nasalized vowels for V_2_ and V_3_ only appeared in plural forms; (ii) nasalized vowels appeared in such a way as to suggest rightward spreading nasal harmony from V_1_, which was always nasalized in plural forms; (iii) this rightward spreading was blocked by different classes of segments in the natural pattern and unnatural pattern groups. In the natural pattern group, stops functioned as blockers: the presence of an intervening stop (i.e., [t, k]) blocked the spreading of nasalization to vowels to the right of the stop. Intervening glides did not prevent rightward spreading of nasalization in the natural pattern group. In the unnatural pattern group, glides functioned as blockers: the presence of an intervening glide (i.e., [w]) blocked the spreading of nasalization to vowels to the right of the glide. Intervening stops did not prevent rightward spreading of nasalization in the unnatural pattern groups. Examples for both patterns can be found in Table 5.

For each of the two patterns, 16 items were generated for presentation during the exposure phase, each item consisting of a singular-plural pair as described above. Items were presented along with pictures illustrating the meaning of the items; for example, a singular item presented as meaning ‘mirror’ would be presented alongside a picture of one mirror, and its corresponding plural item would be presented alongside a picture of two mirrors. The images were used only to convey the fact that the relevant distinction in meaning was between singular and plural forms. Participants were not asked to recall specific pairings of item and meaning, beyond the meaning expressed by the alternation between singular and plural forms. 

The exposure phase consisted of three blocks of repetitions. In each block, the exposure stimuli whose second syllable contained a blocker (8 items in total) were shown once, while the exposure stimuli whose second syllable contained a target (8 items in total) were shown twice. This was done to increase participants’ exposure to items which show nasal spreading: stimuli whose second syllable contained a blocker would show no nasal spreading at all. In total, each item containing a blocker in the second syllable was shown three times and each item containing a target in the second syllable was shown six times, yielding a total number of 72 presentations of stimuli (3 × 8 items with blockers in the second syllable + 6 × 8 items with targets in the second syllable). Within each block, stimuli were presented in a randomized order. Each exposure consisted of: (1) a picture of a single object accompanied by an auditory presentation of the grammar’s singular form, followed by (2) a picture of that same object duplicated side by side accompanied by an auditory presentation of the grammar’s plural form.

In the test phase, items were presented in their singular form along with a pair of possible plurals. One plural form corresponded to the natural pattern grammar and the other to the unnatural pattern grammar. The correct answer depended on what grammar the participant was trained on in the exposure phase: e.g., for the natural pattern group, the answer corresponding to the natural pattern grammar was correct. The test items are presented in their entirety in Table 6 and Table 7.

All 14 test items were repeated four times each, yielding 56 pairs in total. The order in which the items appeared was randomized. Each item was presented to participants auditorily. During the test phase, they were first presented auditorily with a singular form (e.g., [awata]) and were then presented with two possible plural forms. They were asked to choose the form that obeys the plural rule they learned in the exposure phase. If they thought the first test item was correct, they were instructed to press “1”; if they thought the second one was correct, they were instructed to press “2”. For example, a participant exposed to the natural pattern grammar would be presented first with [awata] and then with [ãw̃ãta] and [ãwata]. The participant would be asked to choose which of [ãw̃ãta] and [ãwata] corresponded to the plural rule they had learned in the exposure phase. In this example, [ãw̃ãta] was considered a correct answer for the natural pattern but a wrong answer for the unnatural pattern. 

The items were divided into three categories: previously heard items, new-vowel items, and new-consonant items. Previously heard items are items that had been shown as stimuli in the exposure phase. New-vowel items are items that matched previously heard stimuli where one or both of V_2_ and V_3_ was replaced with [e]. New-consonant items are items that matched previously heard stimuli where one or both of C_1_ and C_2_ was replaced with [k]. The items were not presented along with images during the test phase, because associating the new items with specific images could introduce confounding factors (See [32,33] for more discussion of morphological and semantic effects, such as word frequency or familarity).

### 2.5. Procedure

The experiment consisted of three phases: an exposure phase, a test phase, and a post-test. In each phase, E-Prime was used to present stimuli and record responses. In the trained groups, participants were divided into two groups corresponding to the two artificial grammars, with one group exposed to the natural grammar and the other to the unnatural grammar. Participants in the control group were also divided into two conditions, one exposed to a natural pattern and the other to an unnatural pattern. 

For the trained groups, the exposure phase consisted of three blocks of repetitions. Within each block, tri-syllabic stimuli were presented in a randomized order. As discussed in Section 2.4.2, each exposure consisted of: (1) a picture of a single object accompanied by an auditory presentation of the grammar’s singular form, followed by (2) a picture of that same object duplicated side by side, accompanied by an auditory presentation of the grammar’s plural form. They were told that every singular form is the base form of its corresponding plural form and were instructed to figure out what might govern the relationship between singular and plural forms. Participants could press the Enter key to advance from one form to the next, so that they could control the pace of exposure. The control groups followed the same repetitions, except that only bi-syllabic auditory stimuli were presented, with no pictures given. 

In the test phase, participants in both trained and control groups were presented with a series of tasks. Each task consisted of an exposure to an auditory stimulus which participants were told represented a singular form. Participants were then presented with two alternative plural forms and were asked to select the plural form corresponding to the grammar to which they were exposed during the exposure phase. Note that since no pairing of singular and plural forms were given to the control groups, they were instructed to select the plural form which they thought best resembled the artificial language they heard in the exposure phase based on their intuition. The stimuli consisted of a set of 14 items presented in random order, and the whole set presented 4 times, yielding a total of 56 tasks.

In the post-test phase, participants were presented with a nasalized monosyllable and its oral counterpart. The task was to determine which one was nasalized. All the monosyllables chosen were the ones which were concatenated to create the tri-syllabic stimuli for the exposure and test phases. The purpose of this was to confirm that participants were able to distinguish nasalized monosyllables from their non-nasalized counterparts. This is crucial because if participants could not distinguish nasality at all, we could not be certain that participants were aware that nasal spreading was triggered by the trigger plural marker /ã/ and that nasalization was phonemic in the artificial grammar language they were learning. By this logic, those who failed to distinguish between the trigger /ã/ and its non-nasalized counterpart would be eliminated from the analysis; however, as mentioned in Section 2.3, no exclusions were made on this basis. In addition to the requirement of being able to distinguish the trigger from the non-trigger, an exclusion criterion of below 75% overall correct rate was set. None of participants were excluded based on this criterion. A post-interview was given to probe what learning strategies participants had used to see if they would be able to state any generalizations governing the nasal harmony alternations explicitly. None of the participants was able to state generalizations/rules governing nasal harmony explicitly.

## 3. Results

**Figure 1:**
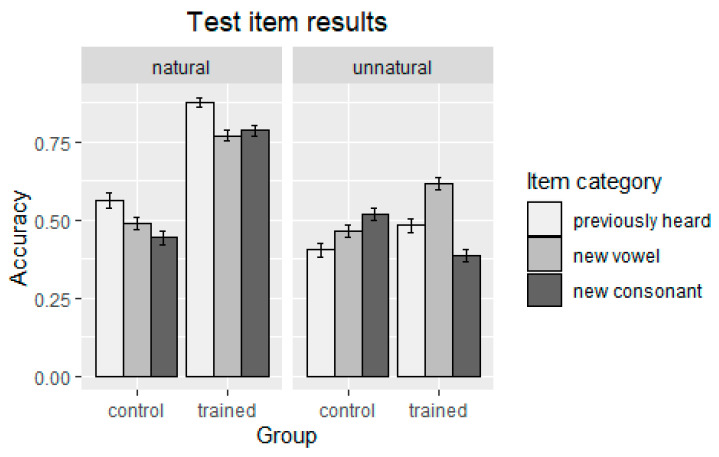
Accuracy of responses to test items.

Participants’ judgment data for the test items (i.e., 56 responses per participant) were analyzed using mixed-effect logistic regression modeling (GLMM), as implemented in the R package lme4 [34] in R version 4.1.1 (R Core Team, 2021 [35]). The dependent variable was accuracy (1 for a correct response on a grammaticality judgment task, 0 for an incorrect response). The fixed predictor variables were ‘naturalness of pattern’, ‘training effect’, ‘item category’, and the interaction between naturalness of pattern and item category. Naturalness of pattern had two levels, coded as ‘natural ‘and ‘unnatural’. The training effect had two levels, coded as ‘trained’ and ‘control’. The test items were divided into three categories: (i) old items heard in the exposure phase (coded as ‘Previously heard’), (ii) new items that differed from items heard in the exposure phase by one vowel (coded as ‘New vowel’), and (iii) new items that differed from items heard in the exposure phase by one consonant (coded as ‘New consonant’). The results are summarized graphically in Figure 1.

Three models of comparison between groups were adopted to verify: (i) within the trained groups, whether the natural pattern was learned better than unnatural patterns, as was predicted; (ii) whether training on harmony patterns affected learning performance, in particular, whether there was a learning difference between the natural trained group and the natural control group; and (iii) whether there was a learning difference between the trained group exposed to the unnatural pattern and the control group exposed to the unnatural pattern. Post-hoc tests were performed to check the degree to which learning occurred for each of the four groups (i.e., those exposed to a natural vs. unnatural pattern × trained vs. control): if the accuracy rate for a group was significantly different from chance (50%), we conclude that learning of some sort, either facilitatory (> 50% accuracy) or inhibitory (<50% accuracy), occurred in that group. The complete R code for the statistical analysis is provided in Appendix A.

The rationale behind choosing a mixed effect logistic regression model is that this model allows us to fit more than two factors and to mix a binary dependent variable with nominal and numerical independent variables, which ANOVA cannot do. Instead of comparing the averaged results by conditions, this model considers individual data points by participant, which the author believes may reflect any effects more precisely. Post-hoc tests were then conducted to see if there were significant learning differences between groups and whether the accuracy rate for a group was significantly different from chance (50%).

### 3.1. Learning of Natural vs. Unnatural Pattern in Trained Groups

To answer the first question, that is, to see whether the trained group exposed to the natural pattern learned their pattern better than the trained group exposed to the unnatural pattern, the group exposed to the unnatural pattern was treated as the reference group. A positive effect of naturalness of pattern would be expected if it were easier for participants to learn the natural pattern than the unnatural one. If the effect of memory alone was involved, with no generalizations depending on naturalness of pattern, a main effect of item category would be expected, with no interaction between pattern of naturalness and type category. Since participants had been familiarized with the old items in the exposure phase, learning relying on the effect of memory alone should not lead to any difference in generalizability between natural and unnatural patterns. The model of the by-subject-and-by-item analysis is shown in Table 8. 

**Figure 2:**
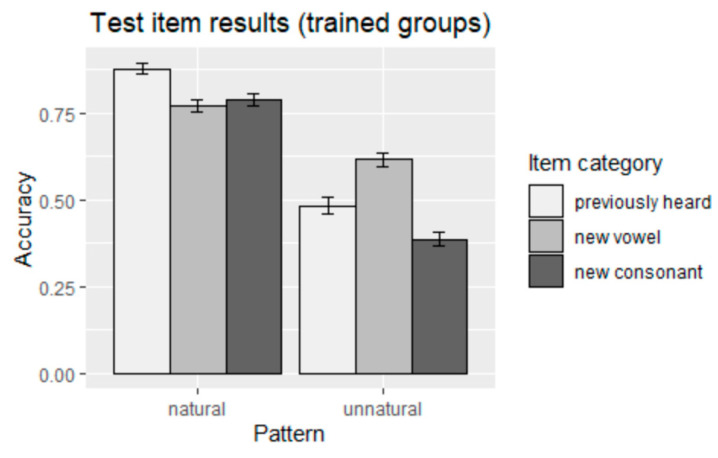
Accuracy of responses to test items (trained groups).

The main effect of naturalness of pattern was positive, suggesting that participants learned significantly better in the natural, sonority hierarchy-obeying nasal harmony pattern than the unnatural, sonority hierarchy-disobeying pattern (*p* < 0.001) (see the findings of the two trained groups in 3). Apart from the main effects of naturalness of pattern (natural vs. unnatural), there was also a main effect of item category (previously heard, new vowel, new consonant) (*p* < 0.001) and an interaction effect between naturalness of pattern and item category (*p* < 0.001). The results for the trained groups are summarized graphically in Figure 2. 

Since the interaction between naturalness of pattern and item category was significant, a post-hoc analysis for item category was conducted using the *emmeans* function, as implemented in the R package *emmeans* [36] with Tukey corrections (confidence level of 0.95). The post-hoc analysis was conducted separately for the natural trained and unnatural trained groups to untangle the nature of the interaction within each group. The results for the natural group showed that the learning of previously heard items was significantly better than that of new-consonant items (*p* < 0.001), and that the learning of previously heard items was significantly better than that of new-vowel items (*p* < 0.001). Neither of these findings was surprising; since previously heard items were the items that participants were presented with in the exposure phase, they were expected to be the easiest to learn. 

The results for the unnatural group, by contrast, showed that the learning of new-vowel items was significantly better than the learning of new-consonant items (*p* < 0.001), and, most surprisingly, that the learning of new-vowel items was significantly better than that of previously-heard items (*p* < 0.001). Both of these differences are contrary to what was found for the groups exposed to the natural pattern, where the categories of new-vowel and new-consonant items were learned equally well, and where previously heard items were learned best of all. 

### 3.2. Learning of the Natural Pattern in Trained vs. Control Groups

To answer the second question, that is, to see whether the better learning performance of the trained group exposed to the natural pattern was due to the natural, sonority hierarchy-obeying nasal harmony pattern they were presented with in the exposure phase, the control group exposed to the natural pattern was treated as the reference group. A positive effect of training would be expected if the exposure of the sonority hierarchy-obeying nasal harmony pattern did facilitate the learning performance. A positive interaction between training effect and type categories would also be expected if (i) the exposure of nasal harmony did facilitate the learning performance of the trained natural pattern and (ii) no pre-existing language-specific bias existed that could somehow influence the control natural group to favor the natural pattern over the unnatural pattern in the test phase. The model of the by-subject-and-by-item analysis is shown in Table 9. 

**Figure 3:**
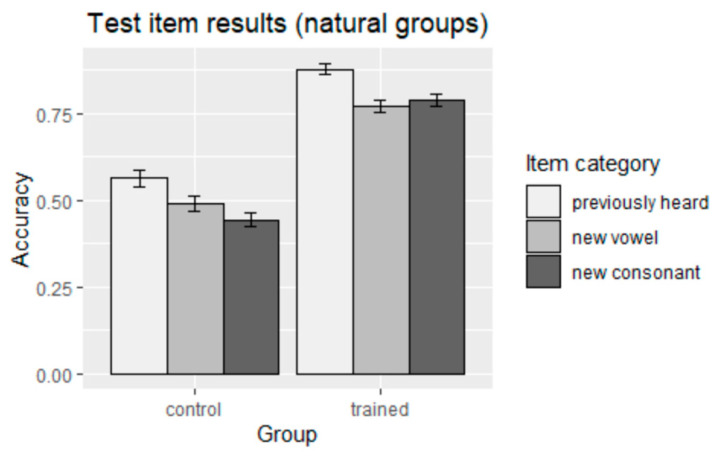
Accuracy of responses to test items (natural groups).

The main effect of training was positive, suggesting that the sonority hierarchy-obeying harmony pattern presented in the exposure phase did facilitate learning (*p* < 0.0001) (see the findings for the two natural groups in 4). As with the trained groups, there was also a main effect of item category (*p* < 0.001) and an interaction effect between naturalness of pattern and item category (*p* < 0.001). The results for the natural groups are summarized graphically in Figure 3. 

A post-hoc Tukey test was conducted to examine whether the training effect could be described as learning: i.e., whether the resulting accuracy was significantly different from chance (50%). Similarly, given the main effect found above, the post-hoc tests could show whether any learning occurred in the control group, even if it had been at a lower level than in the trained group. Specifically, the post-hoc tests checked whether the correct response accuracy (coded as 1) reached an above-chance level, which was an indicator that participants had learned the pattern. The results showed that the natural trained group did learn the pattern (*β* = 2.0155, *SE* = 0.3367, *z* = 5.986, *p* < 0.0001), whereas the natural control group did not (*β* = −0.009226, *SE* = 0.112659, *z* = −0.082, *p* = 0.935). In addition to the main effect of training, there was also an interaction effect between training and item category, where the trend (indicated in the findings for the groups exposed to the natural pattern in 4) showed that all three item categories were learned much better in the trained group than in the control group. 

### 3.3. Learning of the Unnatural Pattern in Trained vs. Control Groups

To answer the third question, that is, to see whether there was a learning difference between the trained group exposed to the unnatural pattern and the control group exposed to the unnatural pattern, the control group was treated as the reference group. If we did find an effect, we could conclude that the pattern is learnable, although perhaps difficult to learn. If, however, no effect of training was found, we might conclude that the pattern is unlearnable. However, a null effect of training might obscure the presence of learning of the pattern if the direction of the training effect were different in different item categories; facilitatory and inhibitory effects existing in different item categories could cancel each other out, yielding no overall effect. For example, training on the unnatural pattern might facilitate accuracy in generalizing the pattern to items with new vowels but inhibit accuracy in generalizing the pattern to items with new consonants. To distinguish between the case where the pattern is unlearnable overall and the case where the pattern is learnable only, e.g., with new vowels, we can look to the interaction between training and item category. If such an interaction effect existed, we could conclude that learning of the pattern is present in some item categories. The model of the by-subject-and-by-item analysis is shown in Table 10. 

**Figure 4:**
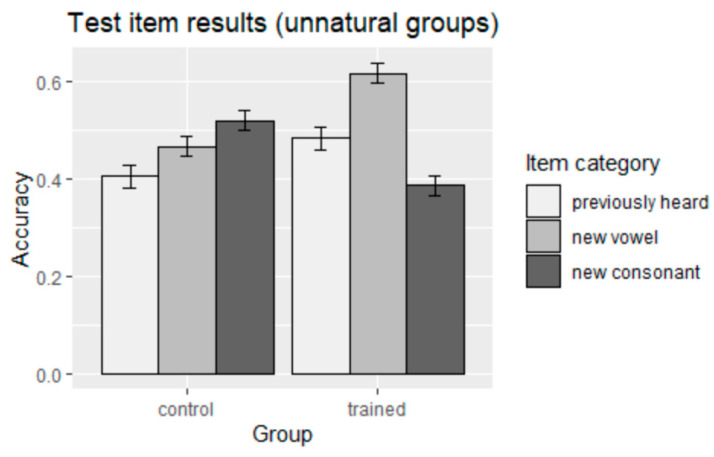
Accuracy of responses to test items (unnatural groups).

No main effect of training was found, suggesting that there was no significant learning difference between the unnatural trained group and its corresponding control group. However, the interaction between training effect and item category was found to be significant (indicated in the findings of the trained and control groups exposed to the unnatural pattern in 5), showing that the accuracy of the unnatural trained group was higher than its control counterpart in previously heard and new-vowel items, and that the trend was flipped for new-consonant items. The results for the unnatural groups are summarized graphically in Figure 4. 

To examine further whether any real learning was involved in either group, the same post-hoc tests done for the natural patterns (i.e., trained vs. control) were adopted to check whether response accuracy (coded as 1) was significantly different from chance, which would serve as an indicator that participants had learned any pattern at all. The results showed that neither the trained group (*β* = 0.007416, *SE* = 0.259664, *z* = 0.029, *p* = 0.977) nor the control group (*β*= −0.1471, *SE*= 0.1655, *z* = −0.889, *p* = 0.374) learned the unnatural pattern. 

In brief, given the answers to our three questions, several implications can be drawn. The fact that the natural pattern was learnable in all three item categories only under training and not by the control group suggests two things. First, it suggests that exposure to the sonority-hierarchy obeying pattern facilitated learning. Second, it suggests that there existed no pre-existing language-specific bias which would facilitate participants’ learning the natural pattern without exposure to it. 

The fact that the group trained on the natural grammar performed better on previously heard items than on items requiring generalization (indicating some effect of memorization), and that their performance after training was above chance, indicates an effect of training. The group trained on the unnatural grammar, by contrast, showed a different pattern of learning; after training, participants performed better on the new-vowel item category than on even previously heard items, although, considering all item categories together, this group’s performance did not differ from chance. This is a curious result, which indicates that perhaps *some* learning did occur in the group trained on the unnatural pattern. With additional time spent in the experiment, the nature of this learning might be elucidated. (In the literature on phonological artificial grammar learning, the length of training is typically less than 30 min (as described in one review article [37]). The shortest length of training reported is 4 min (for palatalization [38]). In the current study, the duration of training was around 20 min for the control groups and around 30 min for the trained groups. One study showed results contradictory to the current study: Lin (2010) [39]. In Lin’s study, unnatural patterns were found to be learned better than natural patterns (Experiments 4, 5). The length of training was around 25 min, which was similar to the current study. However, the design of Lin’s study was fundamentally different from the current study, given that participants in Lin’s study were exposed to both natural and unnatural patterns in the training, making it hard to compare the results to those of the current study. That is, it is hard to determine whether participants in Lin’s study were biased towards, or against, natural patterns, given that they were exposed to both natural and unnatural patterns in the training phase.)

More research is required to determine whether unnaturalness of grammar merely inhibits learning of these unnatural patterns, or whether it truly makes these patterns unlearnable. Nevertheless, the fact that there exists a significant difference between the performance of the groups exposed to the natural and the unnatural grammar given the time allotted to the experiment indicates that there is an effect of naturalness of grammar on learning. (Interestingly, Avcu (2019) [40] found a similar difference in the learning of sibilant harmony patterns in a series of artificial grammar experiments where behavioral measurements were combined with EEG: when participants made a judgment about a natural pattern of sibilant harmony, their brain responses were activated. However, when they made a judgment about an unnatural pattern of harmony, their brain responses were not activated, even though the behavioral results showed some learning. On this basis, the author argues that natural patterns of harmony are learned through domain-specific mechanisms, while unnatural patterns are learned through domain-general mechanisms. Exploring neuroimaging techniques to see if these results replicate for the case of natural and unnatural patterns of nasal harmony is a promising direction for future research.)

## 4. Discussion

Previous research [41] has studied whether natural grammars were preferentially learned over unnatural grammars given ambiguous data. In this paper, we have sought to ascertain whether unnatural grammars are learnable at all, even when given unambiguous data that is incompatible with a natural grammar. The answer is no: phonologically unnatural patterns were not learnable, at least not in the same way or to the same degree as phonologically natural patterns. This finding is in accord with the predictions of phonological theory. Phonologically unnatural patterns are predicted to be not easily learnable in theory as a result of phonological learning biases against such patterns, whether these biases are constraints imposed by UG or arise as a result of the human cognitive or physiological endowment. 

Specifically, these biases come in two types: (i) structural complexity bias, whereby rules involving a greater number of features are harder to learn; and (ii) phonetic substance bias, whereby phonologically unnatural or phonetically ungrounded rules are harder to learn. Using artificial grammar learning, we can investigate the operation of these biases. Thus far, the artificial grammar learning literature has mainly focused on showing the effects of structural complexity bias [42]. Phonetic substance bias has been studied less and with mixed results [37].

Outside of the artificial grammar learning literature, however, many phonetically driven accounts of phonological patterns have been proposed, e.g., by Mielke (2008) [43], Blevins (2004) [44] and Ohala (1993) [45]. This style of explanation can be differentiated from that offered by phonologically driven accounts, which invoke bias facilitated by cognitive dispositions [46]. Each of these forms of bias corresponds to a different idea of ‘naturalness’. Accounts where ‘naturalness’ is defined emergently emphasize that patterns of natural and unnatural classes are emergent from the biased transmission of speech sounds, given the facts of the human speech production and perception systems. That is, as a result of phonetically systematic errors in transmission, certain sound patterns recur across languages. These patterns are then interpreted in terms of phonological features; but, because the patterns are not always identical, the feature specifications themselves are not always identical, but rather homologous [47]. (For example, some natural classes which are phonetically ambiguous, such as [continuant], can pattern differently from language to language. In other words, a given segment may be treated either as [+continuant] or as [-continuant], depending on the language. This ambivalence arises from the fact that phonetic correlates of [continuant] could be defined articulatorily in multiple ways [48].)

In this study, we investigate the effects of one proposed type of phonetic substance bias: the bias against patterns that violate the sonority hierarchy. The current results add another piece of evidence to the study of harmony, namely, that phonologically natural or phonetically grounded harmony rules are favored by phonetic substance bias. (This finding echoes the results of previous studies, which have found that the learning of vowel harmony was significantly better than the learning of disharmony. Vowel harmony and vowel disharmony are structurally comparable, but only vowel harmony is phonetically grounded (co-articulation), and is much more common typologically than the phonetically ungrounded pattern of vowel disharmony [49,50].) It is important to study the effects of phonetic substance bias separately from those of structural complexity bias because only phonetic substance bias is unambiguously specific to language. Structural complexity bias, by contrast, can plausibly be considered a domain-general learning bias, as it acts similarly to other learning biases active outside of the language-exclusive modality [51]. For instance, visual pattern learning also appears to involve a structural complexity bias, in that visual patterns involving a greater number of colors, shapes, or geometrical figures are harder to learn than those involving fewer [42]. Only phonetic substance bias can be used to argue for domain-specific mechanisms in phonological learning [37]. (This distinction between domain-general and domain-specific mechanisms follows the point of view of Moreton and Pater (2012) [37]. Specifically, ‘domain-general’ mechanisms are those that could be used for both linguistic and non-linguistic tasks, whereas ‘domain-specific’ mechanisms are those that are only applicable to linguistic tasks, such as those involving phonetic substance. The example used by Moreton and Pater [37] to illustrate this difference is featural complexity/number of features. They claim that the mechanism used to calculate the complexity of features in phonology is similar to the mechanism that handles categorization of geometric object by shape, color and size (see [42] for more discussion). From this point of view, domain-specific mechanisms are required to explain patterns that are phonetically motivated.) Moreover, structural complexity bias has been shown to admit counterexamples, such as one case where a pattern involving only two features was harder to learn than a three-feature pattern [52]. This potential confounding factor is avoided in the case of phonetic substance bias, because the alternatives being tested all share the same number of features. Therefore, any learning asymmetry that happens must be ascribed to phonetic substance bias.

The sources of phonetic substance bias, however, come in different flavors. Those relevant to nasal harmony are: (i) purely phonetic coarticulation effects, (ii) effects/processes driven by the acoustic correlates of nasal flow and (iii) phonetically grounded phonological processes. In the cases of coarticulation and acoustic correlate effects, no appeal to phonology is necessary. Nasal harmony, however, cannot be fully explained either as coarticulation, i.e., a persistence of the articulatory gesture of lowering the velum past the end of the trigger consonant, or as an effect of the acoustic correlates of nasal flow. If the phonetic effects of articulation and perception sufficed as an account of nasal harmony, we would be unable to explain nasal harmony patterns involving transparent segments, where segments that bear no nasal airflow (e.g., glottal stops) nevertheless allow nasal harmony to pass through them. Various accounts of this behavior have been given, including by [7], who treats transparent segments such as glottal stops as phonetically non-nasal because of a lack of nasal airflow, but phonologically still targets/undergoers of nasal harmony. Cohn (1993) [14], however, found that transparent glottal stops are phonetically (i.e., articulatorily) nasal because the velum is still lowered in their production, even though no nasal airflow is present. The debate over how exactly to account for transparent segments in nasal harmony has not yet come to an agreement. However, crucially, if nasal harmony were simply an epiphenomenon of phonetic factors, nasal harmony with transparent segments would not be expected. Nasal harmony must be explained as a phonological pattern, one that interacts with the sonority hierarchy. (Note that the nasalized segment hierarchy is not identical to the sonority hierarchy when glottals are considered. Glottals have been argued to function either as transparent segments or ungergoers of nasalization. However, the segments of interest in the current study are stops ([t], [k]), glides ([w]), and vowels. Without glottals, the nasalized segment hierarchy is equivalent to the sonority hierarchy.)

Phonetically grounded phonological patterns are usually reflected in typological tendencies. Natural patterns appear across the world’s languages with much higher frequency than unnatural patterns. Nasal harmony is interesting in this regard because patterns of nasal harmony that do not conform to the nasal segment hierarchy are not merely uncommon but have not been found at all. For this reason, we may be especially tempted to propose that this state of affairs is a result of some aspect of the human cognitive or physiological endowment. The value of artificial grammar learning is that it provides a way to examine this question more directly than the typological route, which is plagued by the effects of contingency in history. (One attempt to explain the relationship between emergent theories of naturalness and diachronic change is that typologically common patterns can be derived from different processes from typologically rare patterns. Michaud et al. (2012) [53] have proposed that typologically common patterns are those that can be derived from synchronically natural (in the sense above of ‘phonetically systematic errors in transmission’) phonological patterns; that is, they can be explained with reference to biases in production and perception of speech sounds. An example of this type of pattern would be C-to-V nasalization, which is straightforwardly explicable in terms of coarticulation. Typologically rare patterns, on the other hand, require an appeal to diachrony.)

Moreover, it is not possible to find two natural languages that differ *only* in whether a particular set of segments act as transparent or opaque. Adopting the artificial grammar paradigm, however, allows us to do so. Specifically, in the current study, we examined two artificial grammars, one that generated test items that reflected the sonority-obeying hierarchy, and one that generated test items that reflected a sonority-disobeying hierarchy. Since stops such as [t, k] would not have nasal airflow when they served as targets for nasal harmony (in the grammar conforming to the sonority-disobeying hierarchy), technically the stops /t, k/ were transparent to [nasal] in that they did not block nasal harmony. However, /t, k/ were opaque to nasal harmony when they served as blockers for nasal harmony (in the grammar conforming to the sonority-obeying hierarchy), given that there was no nasal flow for [t, k] and they blocked nasal harmony. The prediction that the learning difficulty of the sonority-disobeying pattern would be higher than that of the sonority-obeying pattern was supported by the current findings. The sonority-disobeying pattern was not learnable in the same way as the sonority-obeying pattern; even after training, participants could not produce the unnatural pattern at an accuracy level distinguishable from chance.

Even though the artificial grammar paradigm often arouses the criticism that it lacks the ecological validity of natural language acquisition, it is a beneficial paradigm in many ways. First, it is hard to tease apart some of the entangled factors involved in natural languages, but it is easy to tease apart and manipulate factors by using an artificial grammar learning paradigm. The current study aims to isolate the effects of training, naturalness of pattern, and item category. Second, the paradigm enables the experimenter to design an artificial grammar with an appropriate level of difficulty [49]. That is, the grammar can be relatively simple but hard enough to allow the experimenter to tease apart which factors are language-specific and which factors are universal. Third, unrelated factors can be easily avoided. For instance, in artificial grammar learning, one does not need to worry about the influence of frequency effects from real language. Lexical semantic effects can also be avoided if the experimenter is only interested in the relationships of sounds. Finally, using this paradigm is economical in the sense that one only needs to test one language population rather than many, based on the assumption that learning biases are shared by all humans [50]. 

All in all, the current findings are of value in two ways. First, this study differs from most previous works in that it investigates a harmony pattern rather than a phonotactic rule. Unlike phonotactic rules, harmony patterns cannot be learned on the basis of transitional probabilities. Instead, the learning of harmony patterns involves a complicated process whereby a trigger spreads a feature to targets and halts when encountering blockers. Harmony is an iterative process and can only be captured when we consider a mapping between underlying and surface representations in a stepwise fashion, at least in a derivational framework. Previous artificial grammar studies [52] have tried to test the mapping between underlying and surface representations or to test the effect of the sonority hierarchy in consonant cluster phonotactics [54]. Few studies [32,55], however, have tested the interaction between representations and phonetically grounded hierarchies involving multiple segments. Second, in examining nasal harmony, this study has tested not only the mapping between underlying and surface representations, but also a pattern which involves dependencies between consonant and vowel representations. Patterns involving dependencies between vowel and consonant features (represented using separate vowel and consonant tiers) have been argued to be more difficult to learn than patterns involving only consonant or only vowel features [46]. The difficulty of patterns which require reference to both consonant and vowel tiers has been shown by [46]. This study found that voicing-height harmony was harder to learn than patterns involving only consonantal or vowel tiers, such as voicing agreement [46]. Other commonly studied harmony patterns, such as sibilant harmony [24,56,57,58] or vowel harmony [50,59], only involve one tier. Nasal harmony, by contrast, involves both, and therefore presents an opportunity for us to study the factors that facilitate learning even of difficult patterns. 

This study examined the effect of a phonological naturalness constraint on nasal harmony grammars in facilitating learning of nasal harmony. The constraint in question is the nasalized segment hierarchy, an application of the well-attested sonority hierarchy. If a difficult pattern such as nasal harmony is still learnable in the laboratory, but only in the case where the harmony pattern proceeds according to phonologically natural rules, this is good evidence for the crucial role of phonological naturalness in learnability.

## 5. Conclusions

The current study showed that the sonority hierarchy-obeying pattern was learnable but the sonority-disobeying one was not, at least not in the same way or to the same degree. Nasal harmony is a thorny pattern because which elements come from universals and which elements come from language exposure is debated. There is also a debate about what should be explained in terms of phonetic substance, and what should be explained by phonology. The phonological operation of nasal harmony involves iterative feature spreading across consonant and vowel tiers and cannot be fully explained by transitional probabilities in phonotactics or by using a one-tier theory. Overall, the findings showed that the natural, sonority-obeying pattern is learnable, whereas the unnatural, sonority-disobeying pattern is unlearnable or less learnable. This learning asymmetry cannot be ascribed to structural complexity bias, but rather must be the result of phonetic substance bias, which is domain-specific to natural language acquisition. 

## Figures and Tables

**Table 1:** The consonant inventory of Min in IPA (Following [27]).

	Bilabial	Labiodental	Dental	Alveolar	Postalveolar	Retroflex	Palatal	Velar	Labial-velar	Glottal
Stop	p p^h^ b			t t^h^ l				k k^h^ ɡ		ʔ
Nasal	m			n				ŋ		
Fricative				s z						h
Affricate				ts ts^h^						
Approximant							j		w	

**Table 2:** Phonemic vowels in Min (Following [27]).

	Front	Central	Back
High	i ĩ		u
High-mid	e ẽ		o
Low-mid			ɔ ɔ̃
Low		a ã	

**Table 3:** Predictions concerning the generalizability of natural and unnatural patterns.

	Exposure	Test	Prediction
Natural group	more sonorous: target [w] less sonorous: blocker [t]	more sonorous: target [w] new segment [k]: same class as blocker	new segment [k] is blocker
Unnatural group	more sonorous: target [t] less sonorous: blocker [w]	more sonorous: target [t] new segment [k]: same class as target	not generalizable to [k] due to the violation of the implicational universals

**Table 4:** One set of designs for exposure stimuli for control groups.

Natural Pattern (Sonority Hierarchy Pattern)	Unnatural Pattern (Reverse Sonority Hierarchy Pattern)
[tawa] [wata] [tata] [wawa]	[tawa] [wata] [tata] [wawa]

**Table 5:** One set of designs for exposure stimuli for trained groups.

Natural Pattern (Sonority Hierarchy Pattern)		Unnatural Pattern (Reverse Sonority Hierarchy Pattern)		Picture (‘Meaning’)
singular	plural	singular	plural	singular vs. plural
[atawa] [awata] [atata] [awawa]	[ãtawa] [ãw̃ãta] [ãtata] [ãw̃ãw̃ã]	[atawa] [awata] [atata] [awawa]	[ãtãwa] [ãwata] [ãtãtã] [ãwawa]	‘mirror’ vs. ‘mirrors’ ‘car’ vs. ‘cars’ ‘table’ vs. ‘tables’ ‘cup’ vs. ‘cups’

**Table 6:** Test stimuli presented to the group exposed to the natural pattern.

Item Category	Singular	Possible Plural 1 (Pattern-Obeying)	Possible Plural 2 (Pattern-Disobeying)
Previously heard	[awata]	[ãw̃ãta]	[ãwata]
	[atawa]	[ãtawa]	[ãtãwa]
	[awawa]	[ãw̃ãw̃ã]	[ãwawa]
	[atata]	[ãtata]	[ãtãtã]
New vowel	[aweta]	[ãw̃ẽta]	[ãweta]
	[atawe]	[ãtawe]	[ãtãwe]
	[awate]	[ãw̃ãte]	[ãwate]
	[awewe]	[ãw̃ẽw̃ẽ]	[ãwewe]
	[atewa]	[ãtewa]	[ãtẽwa]
New consonant	[akawa]	[ãkawa]	[ãkãwa]
	[awaka]	[ãw̃ãka]	[ãwaka]
	[akaka]	[ãkaka]	[ãkãkã]
	[akata]	[ãkata]	[ãk̃ãtã]
	[ataka]	[ãtaka]	[ãtãkã]

**Table 7:** Test stimuli presented to the group exposed to the unnatural pattern.

Item Category	Singular	Possible Plural 1 (Pattern-Obeying)	Possible Plural 2 (Pattern-Disobeying)
Previously heard	[awata]	[ãwata]	[ãw̃ãta]
	[atawa]	[ãtãwa]	[ãtawa]
	[awawa]	[ãwawa]	[ãw̃ãw̃ã]
	[atata]	[ãtãtã]	[ãtata]
New vowel	[aweta]	[ãweta]	[ãw̃ẽta]
	[atawe]	[ãtãwe]	[ãtawe]
	[awate]	[ãwate]	[ãw̃ãte]
	[awewe]	[ãwewe]	[ãw̃ẽw̃ẽ]
	[atewa]	[ãtẽwa]	[ãtewa]
New consonant	[akawa]	[ãkãwa]	[ãkawa]
	[awaka]	[ãwaka]	[ãw̃ãka]
	[akaka]	[ãkãkã]	[ãkaka]
	[akata]	[ãk̃ãtã]	[ãkata]
	[ataka]	[ãtãkã]	[ãtaka]

**Table 8:** Summary of the effects in the experiment (trained groups).

	Chisq	Df	Pr (>Chisq)
Naturalness of pattern	38.432	1	< 0.001 ***
Item category	37.008	2	< 0.001 ***
Naturalness of pattern × Item category	56.163	2	< 0.01 **

Signif. codes: ‘***’ 0.001 ‘**’ 0.01.

**Table 9:** Summary of the effects in the experiment (natural groups).

	Chisq	Df	Pr (>Chisq)
Training effect	61.4366	1	< 0.001 ***
Item category	33.9480	2	< 0.001 ***
Training effect × Item- category	7.0437	2	< 0.05 *

Signif. codes: ‘***’ 0.001 ‘*’ 0.05.

**Table 10:** Summary of the effects in the experiment (unnatural groups).

	Chisq	Df	Pr (>Chisq)
Training effect	0.7786	1	0.3776
Item category	24.8363	2	< 0.001 ***
Training effect × Item category	54.3586	2	< 0.001 ***

Signif. codes: ‘***’ 0.001.

## Data Availability

The datasets used for the current study are available from the corresponding author.

## Appendix A. Details of Statistical Models Are Provided

(1) Logistic regression model:

Trained groups (natural vs. unnatural)

Accuracy ~ Naturalness * Item category + (1|Subject) + (1|Test item)

Natural groups (trained vs. control)

Accuracy ~ Group * Item category + (1|Subject) + (1|Test item)

Unnatural groups (trained vs. control)

Accuracy ~ Group * Item category + (1|Subject) + (1|Test item)

(2) Post-hoc tests done to check whether response accuracy (coded as 1) was significantly different from chance:

Accuracy ~ 1 + (1|Subject) + (1|Test item)

(3) A post-hoc analysis for item categories was conducted using *emmeans* to untangle the nature of the interaction within each trained group (natural vs. unnatural): emmeans(data, list(pairwise ~ Item category, adjust = “tukey”))
